# Loneliness and Mental Health Disorders in Older Adults Living in Portugal During the COVID-19 Pandemic: A Cross-Sectional Study

**DOI:** 10.3390/healthcare13131483

**Published:** 2025-06-20

**Authors:** Odete Araújo, Lia Sousa, Francisco Sampaio, Cláudia Rodrigues, Nadine Correia Santos, Carlos Sequeira, Laetitia Teixeira

**Affiliations:** 1 School of Nursing, University of Minho, 4710-057 Braga, Portugal; a97348@uminho.pt; 2 Health Sciences Research Unit: Nursing (UICISA: E), Nursing School of Coimbra (ESEnfC), 3045-043 Coimbra, Portugal; 3 Nursing Research Centre, University of Minho, 4710-057 Braga, Portugal; 4 Vale do Ave Higher School of Health, 4760-409 Vila Nova de Famalicão, Portugal; lia.sousa@ipsn.cespu.pt; 5 CINTESIS@RISE, Nursing School of Porto (ESEP), 4200-450 Porto, Portugal; franciscosampaio@esenf.pt (F.S.); carlossequeira@esenf.pt (C.S.); 6 Nursing School of Porto (ESEP), University of Porto, 4200-072 Porto, Portugal; 7 Life and Health Sciences Research Institute (ICVS), School of Medicine, University of Minho, 4710-057 Braga, Portugal; nsantos@med.uminho.pt; 8 ICVS/3B’s—PT Government Associate Laboratory, 4710-057 Braga, Portugal; 9 School of Medicine and Biomedical Sciences (ICBAS), University of Porto, 4050-313 Porto, Portugal; laetitiateixeir@gmail.com

**Keywords:** mental health, older people, pandemic, loneliness, depression, anxiety, sleep disorder

## Abstract

**Background/Objectives**: The COVID-19 pandemic has had a profound impact on the mental health of the general population, particularly older adults. This study aimed to explore the association between loneliness and mental health disorders in this demographic during the pandemic. **Methods**: A cross-sectional survey was conducted in Portugal using data from the Survey of Health, Ageing and Retirement in Europe (SHARE) database between June and August 2020, during the COVID-19 pandemic (Wave 8 COVID-19 Survey), using computer-assisted telephone interviews. **Results**: The final sample included 836 participants, with 387 (46.4%) men and a mean age of 74.5 years (SD = 6.7). Mental health indicators revealed that 441 (52.1%) reported feelings of nervousness, 384 (45.3%) experienced sadness or depression, 349 (41.2%) encountered sleeping difficulties, and 280 (33.1%) reported experiencing loneliness often or some of the time. Increased feelings of loneliness were notably associated with women in poorer health, those with heightened fear of falling, dizziness, fatigue, anxiety, depression, and concurrent health and sleep issues. Age and medication use did not significantly impact feelings of loneliness. **Conclusions**: The findings highlight a potential association between adverse mental health outcomes among older adults during the initial phase of the pandemic. Future research, employing longitudinal research designs, is warranted to explore these relationships more rigorously, in a post-pandemic context, and to inform effective intervention development and strategies to prevent mental health problems within this vulnerable population.

## 1. Introduction

Many European countries, along with other regions worldwide, are set to face the challenge of an aging population in the coming decades. Projections indicate that by 2100, over 30% of European citizens will be aged 65 and older [1]. Current estimates predict that 176 million older individuals will reside in European countries, reflecting the global trend of population aging. Over the past five decades, the number of people aged 65 and over has doubled, rising from less than one million to almost 2.5 million in 2022 [2].

Mental health in older adults is a multidimensional concept that transcends the mere absence of psychopathology. According to the World Health Organization [3], it is defined as a state of well-being in which individuals are able to recognize their abilities, effectively manage life’s stressors, engage in meaningful and productive activities, and contribute to their communities. While aging itself is not a problem associated with functional loss, accumulating evidence indicates that advancing age increases the likelihood of chronic diseases [4]. In later life, mental health assumes particular importance, as psychosocial resources become increasingly vital for managing age-related losses [5]. Certain mental health issues, such as depression and anxiety, sleep disorders, cognitive decline, and other mental health conditions, are not exclusive to older adults but are intricately linked to advancing age and are of great concern [6]. Contemporary perspectives conceptualize mental health in older adults as a dynamic interplay between positive indicators, such as well-being, and negative indicators, including depression and cognitive decline. Thus, the promotion of mental health in this demographic requires a dual approach based on the prevention and mitigation of psychopathological symptoms, alongside the active enhancement of psychological well-being [5].

Depression in older adults is a global health issue, with an underdiagnosed prevalence of 4–9% worldwide [7]. This underdiagnosis, combined with inadequate treatment, often results in delayed interventions, consequently increasing mortality and morbidity in this population, with some evidence suggesting a rising incidence [8]. Depression is considered a significant contributor to disability in older people and is frequently associated with cognitive and physical decline, contributing to premature mortality [9]. Another prevalent mental health concern among older adults is anxiety disorders. Authors have identified key factors such as female gender, cognitive and physical frailty, chronic illnesses, poor perception of health status, limited resources, and inadequate coping strategies for complex situations as significant determinants of anxiety disorders in this population [10].

Older adults, characterized as a vulnerable group due to the prevalence of chronic illnesses, limited financial and social resources, and difficulties in participating in social networks, became more susceptible to isolation and loneliness during the COVID-19 pandemic. This new paradigm, with physical distancing becoming a daily reality, had a significant impact on those aged 65 and above. While aging is not a direct determinant of loneliness, some studies suggest that older individuals face multiple losses, including the passing of relatives and friends, requiring greater coping efforts to manage physical and mental challenges [11,12]. The social distance measures imposed during the pandemic also intensified social isolation, particularly among older adults [13]. Recurrent social isolation can exacerbate feelings of loneliness, leading to poorer mental health and overall well-being [14]. Living alone further heightens the high risk of social isolation and loneliness, as well as cognitive decline, increasing the risk of developing dementia in older adults [15]. However, it is important to recognize that social isolation and loneliness are not synonymous—socially isolated individuals are not always lonely, and lonely individuals are not necessarily socially isolated [11,12].

Some studies have demonstrated that the pandemic itself intensified feelings of loneliness among older people, with high levels attributed to social restrictions and the pervasive fear of being infected [11,16,17,18,19]. Thus, the Canadian Longitudinal Study on Ageing (CLSA), led by Susan Kirkland and Lauren Griffith [20], examined the prevalence of loneliness and associated risk factors among older adults during the COVID-19 pandemic. The study included 44,817 community-dwelling older adults before the pandemic (2015 to 2018), with 24,114 participants surveyed during the pandemic between September and December 2020. Results showed that the prevalence of loneliness increased to approximately 50% during the pandemic compared to about 30% before.

Moreover, the COVID-19 pandemic acted as a catalyst for the exacerbation of chronic fatigue, negatively impacting the overall quality of life in older adults [21]. Findings by Aly and Saber revealed significant associations between chronic fatigue syndrome and cognitive impairment, fatigue, sadness, stress, sleep disturbances, and recurrent falls during the post-recovery period [21]. Aging is closely associated with mood alterations, often intricately intertwined with poor sleep quality [22,23,24]. A study by Pires et al. underscored the ongoing lack of evidence regarding sleep patterns in older adults and their potential correlation with the COVID-19 pandemic [25]. Nevertheless, it is widely acknowledged that the pandemic exposed older individuals to additional risks, leading to hospitalization, mechanical ventilation, and, in many cases, death. This exacerbated clinical situation, combined with chronic diseases and frailty, may have induced modifications in sleep architecture, affecting sleep duration and efficiency, thereby increasing the incidence of sleep disorders [25,26].

Prior to the COVID-19 pandemic, Portugal reported a prevalence of depression of about 12% [27]. Additionally, the prevalence of anxiety disorders among older adults in Portugal is approximately 10% [27], a figure notably lower than in other European countries, where prevalence rates among community-dwelling older people range between 15% and 52% [28]. While prior research has analyzed the general impact of pandemic-related loneliness and mental health disorders, few have focused explicitly on Portuguese older adults and considered the enduring consequences beyond the immediate crisis. By examining this demographic, our study provides a unique perspective on the intersection of loneliness, aging, and mental health throughout the initial phase of the pandemic, offering insights that can inform public health policies and targeted interventions for older Portuguese people. Therefore, this study aimed to explore the association between loneliness and mental health disorders in Portuguese older adults during the COVID-19 pandemic.

## 2. Materials and Methods

### 2.1. Study Design and Participants

**Figure 1:**
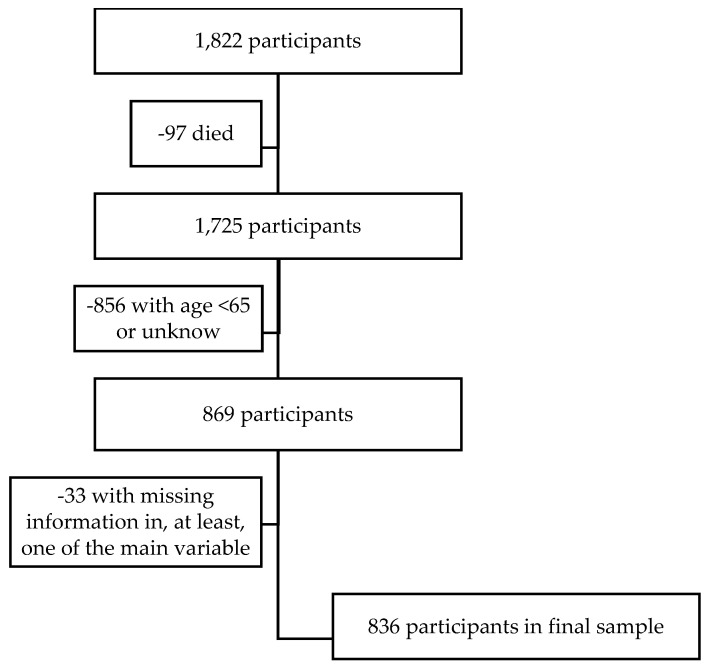
Flowchart of the participants of the study.

Data from the Wave 8 COVID-19 Survey 1, Release version: 8.0.0, of the Survey of Health, Ageing and Retirement in Europe (SHARE) project were used [29]. In Portugal, the survey was conducted from June to August 2020, within panel households, using computer-assisted telephone interviews (CATI) due to the COVID-19 pandemic and the enforcement of lockdown measures [30]. A total of 1822 individuals participated. Study exclusion criteria included participants aged below 65 years or with unknown age (*n* = 856) and those with missing data (*n* = 33) in at least one of the following variables: feeling nervous in the last month, feeling sad or depressed in the last month, trouble sleeping recently, or how often they felt lonely. Consequently, the final sample for this study consisted of 836 participants (Figure 1). Comprehensive methodological information, including sampling procedures and the operationalization of variables, can be found in the *SHARE Corona Survey Release Guide 8.0.0.*

### 2.2. Measures

The survey included basic demographic questions such as age and gender. Additionally, participants were asked about their self-perception of health: “Before the coronavirus outbreak, would you say your health was excellent, very good, good, fair, or poor?”; changes in health since the outbreak: “If you compare your health status with that before the coronavirus outbreak, would you say your health has improved, worsened, or remained the same?”; and whether they had been diagnosed with a major health condition: “Since we last interviewed you, were you diagnosed with a major illness or health condition?”. The list of diagnoses included hip fracture, diabetes or high blood sugar, high blood pressure or hypertension, heart attack or other heart problem, chronic lung disease, cancer or malignant tumor, other illness, or health condition.

Mental health indicators were initially assessed through questions about the use of prescribed medication and sleep quality: “Do you regularly take prescribed medication?” and “Have you had trouble sleeping recently?”. Participants were also asked, “For the past six months, at least, have you been bothered by any of the following health conditions?” with options such as falling more often, fear of falling, dizziness, fainting, blackouts, and fatigue. Finally, mental health disorders and loneliness were evaluated using more specific questions: “In the last month, have you felt nervous, anxious or on edge?”; “In the last month, have you been sad or depressed?”; “How much of the time do you feel lonely? Often, some of the time, or hardly ever or never?”.

### 2.3. Statistical Analysis

Sample characteristics were obtained based on frequencies (absolute and relative) for qualitative variables and mean and standard deviation for quantitative variables. Groups were compared utilizing the Chi-Square test for qualitative variables and independent *t*-tests for quantitative variables. Binary logistic regression models (unadjusted and adjusted multivariable) were performed to identify potential factors associated with the outcomes, “nervous” and “feeling sad or depressed”. In the multivariable model, age (the single quantitative variable) was included as a z-score. Thus, odds ratios could be used as measures of effect size. All variables were binary. Additionally, Cox and Snell R^2^ and Nagelkerke R^2^ were reported. All analyses were performed using the software IBM SPSS version 28.0, with a significance level set at 0.05.

### 2.4. Ethical Considerations

The SHARE study was reviewed and approved by the Ethics Committee of the University of Mannheim, and different waves were reviewed and approved by the Ethics Council of the Max Planck Society and the Ethics Councils of the participating countries. All this information was entered into a database (https://share-eric.eu, accessed on 3 June 2024).

## 3. Results

*Sample Characteristics*

The sample consisted of 836 participants, with 387 (46.3%) men, and an average age of 74.5 years (SD = 6.7 years). Before the COVID-19 pandemic, most participants rated their health as either fair (41.9%) or poor (19.7%), while 106 (12.7%) reported that their health had worsened. Additionally, 163 participants (19.5%) had been diagnosed with at least one major health condition. Frequent falls were reported by 136 participants (16.3%), fear of falling by 336 (40.2%), dizziness, fainting, or blackouts by 238 (28.5%), and fatigue by 402 (48.1%). Nearly all participants were on a regular regimen of prescribed drugs. Regarding mental health, 435 (52.0%) felt nervous, 378 (45.2%) reported feeling sad or depressed, 346 (41.4%) had trouble sleeping, and 279 (33.4%) reported feeling lonely often or some of the time (Table 1).

Gender, health (self-perception and change), fear of falling, dizziness, fainting or blackouts, fatigue, and mental health factors (feeling nervous, sad or depressed, and trouble sleeping) were all associated with feelings of loneliness. Women, as well as participants with poor physical and mental health, were more likely to report feeling lonely often or some of the time. Table 2 outlines the characteristics of the groups defined by feelings of loneliness status.

Gender, age, fatigue, sleeping disorders, and feelings of loneliness were all factors associated with feeling nervous. Specifically, women, younger participants, those experiencing fatigue, sleep problems, and those who often or sometimes felt lonely were more likely to feel nervous. Similarly, in terms of “feeling sad or depressed”, women, individuals diagnosed with a major health condition, those with fatigue, sleep troubles, and those feeling lonely often or some of the time were likely to report feeling sad or depressed. Detailed information on the factors associated with feeling nervous and feeling sad or depressed is presented in Table 3 and Table 4.

## 4. Discussion

The primary findings of this study revealed a significant correlation between heightened feelings of loneliness and women living alone. This demographic exhibits concerning trends, including poorer health statuses and increased vulnerability to various health conditions. Bhat et al. [31] similarly identified that older women were already vulnerable during the COVID-19 pandemic due to factors such as social isolation, persistent health problems, and caring responsibilities. In our study, this group also reported greater concerns about their physical well-being, such as elevated fear of falling, dizziness, fatigue, anxiety, depression, and disturbances in sleep patterns. Prior research suggests that such psychological suffering may stem from prolonged isolation, fear, and the uncertainty that characterized the pandemic [31].

Relevant consideration pertains to the potential confounding effect of age on the outcomes of interest, given that aging is intrinsically associated with a range of physical and mental health challenges. In our study, age was treated as a continuous variable and standardized (z-score) for inclusion in the multivariable logistic regression models. This approach allowed for the estimation of effect sizes through odds ratios, ensuring that age-related variance was accounted for in the statistical models. However, no significant associations between age and the mental health indicators under study emerged from the adjusted analyses. This finding suggests that, within our sample of older adults (≥65 years), differences in mental health outcomes may be more attributable to individual health status and psychosocial factors than to chronological age alone. Nonetheless, the cross-sectional design of the study limits our capacity to assess longitudinal effects of aging or to examine within-subject health changes before and after the onset of the COVID-19 pandemic.

Those living alone may face fewer opportunities for social engagement, which limits the mobilization of cognitive reserves and contributes to cognitive decline/impairment and depression. Furthermore, individuals who are widowed, divorced, separated, or single are more likely to live alone, while marriage or cohabitation offers a protective effect for cognitive health [32,33].

Our research is aligned with previous evidence, which has found that factors contributing to loneliness include being female, living alone, having limited financial resources, having children, and low contact with neighbors [34]. Additionally, the pandemic revealed that women and individuals with lower incomes reported higher levels of loneliness [34], which also corroborated our findings. Another study has found that loneliness was higher in women (22% vs. 17%), those living in urban areas (21% vs. 15%), and lower in individuals aged 75 and over (16% vs. 20% or higher in younger age groups) [20]. The study also highlighted a strong association between pandemic-related loneliness and pre-pandemic loneliness, alongside sociodemographic variables such as living alone, residing in urban areas, depression, having two or three or more chronic illnesses, and health-related behaviors like regular or no alcohol consumption [20]. By fostering social connections, promoting mental stimulation, and establishing robust support systems, the risk of cognitive decline among older individuals living alone can be mitigated, ultimately enhancing their overall cognitive well-being [35].

Several studies have documented an increase in loneliness among older adults since the onset of the COVID-19 pandemic [19,30,36]. Loneliness is recognized as a risk factor for depression, making it essential for health professionals to assess and address signs of loneliness in this population [33]. Recent research further aligns with our results and prior studies that link increased depressive symptoms and anxiety directly to loneliness [12,37,38,39]. Among the various consequences of the pandemic on the physical and mental health of older individuals, sleep disorders have been particularly impactful, exacerbating mental health issues in this vulnerable group [30]. Sleep disturbances increase the likelihood of depression, anxiety, and loneliness [30,32]. Corbo et al. [40] further support this, concluding that individuals diagnosed with mental health disorders are more likely to experience poor sleep quality compared to the general population.

Another outcome of the COVID-19 pandemic was the heightened risk of falling and fear of falling among older adults, a phenomenon linked to prolonged home confinement. This situation led to increased fear of movement, reduced physical activity, and, consequently, a greater risk of falls [41]. The relationship between extended stays at home and diminished physical well-being highlights serious implications for the overall health and safety of older individuals. Fear of movement can discourage engagement in regular physical activities, fostering a sedentary lifestyle that further declines muscle strength, balance, and coordination.

Addressing these challenges is paramount for promoting the health and mobility of older individuals. Interventions that encourage safe, gradual physical activity, cultivate supportive home environments, and tackle the psychological aspects of movement-related fear are essential for mitigating risks and enhancing overall well-being. These findings align with other studies that have reported an increased risk of falling among older adults [42]. Falls are often associated with the social isolation and loneliness that many older individuals experience [43], even when no observed effects on the number of falls are noted, by contributing to increased sedentary behaviors [44].

Chronic fatigue syndrome is a significant concern among older adults, negatively impacting overall health and showing higher prevalence in women [45]. The COVID-19 pandemic has exacerbated this issue due to increased comorbidities and the overall vulnerability of older individuals [46]. The findings from Aly and Saber [20] highlight the relationship between fatigue and cognitive impairment, stress, sadness, sleep disorders, and recurrent falls, with these symptoms being particularly prevalent among women, affecting 33% of respondents.

In the field of gerontological science, human aging as a process requires dynamic systems that include the interactions of biological, psychological, social, economic, and socio-cultural variables. This integrative and multifactorial view contrasts with a view of the biopsychopathological dimension, the paradigm of which persists in many clinical and social contexts. The cognitive functioning of older people varies over time, depending on their cognitive reserve and the impact of other personal, contextual, and behavioral variables, resulting in positive gains such as well-being and resilience or, conversely, losses caused by the confluence of factors such as anxiety, cognitive changes or even loneliness [47].

### 4.1. Limitations

Several limitations of this study should be acknowledged. Firstly, its cross-sectional design restricts our ability to compare variables before and after the COVID-19 pandemic, particularly regarding changes in perceived health. To gain a comprehensive understanding of the evolution of these variables within the general population and, especially among older adults, further research employing different study methodologies is essential. Additionally, using a subsample of a survey limits our ability to claim representative results.

One important limitation of this study concerns the phrasing of a key item used in data collection. Participants were asked: “In the last month, have you been sad or depressed?”. Although this question was designed to broadly capture the presence of low mood, it conflates two distinct emotional and clinical states—sadness and depression—which are not synonymous. Sadness is a normal affective response to adverse events and does not necessarily indicate psychopathology, whereas depression refers to a clinical syndrome that involves a constellation of symptoms, including, but not limited to, persistent low mood, anhedonia, and functional impairment. The conflation of these terms may have led to over- or underestimation of the prevalence of clinically significant depressive symptoms. Therefore, caution is warranted in the interpretation of these findings, as the use of a single, compound question may have limited the specificity and diagnostic validity of the self-reported data.

Therefore, we recommend more in-depth investigations into the COVID-19 pandemic’s impact, specifically regarding its effects on loneliness, sleep, and the physical and mental health of older individuals. Such research could inform the implementation of more effective preventive and global health programs.

### 4.2. Practical Implications

These findings offer valuable insights that can influence clinical practice, particularly in tackling the mental health challenges faced by older individuals in the aftermath of the pandemic. By integrating these insights into clinical practice, healthcare professionals can better support the mental health of older adults amidst the unique challenges posed by COVID-19.

This study contributes to health education by improving the understanding of mental health issues among older people during COVID-19. Potential initiatives could include training programs for identifying indicators of loneliness and encouraging active aging. By incorporating these considerations into health education, educators can foster a community that actively promotes the mental health and overall well-being of older individuals. Finally, the strong association between living alone and various adverse health outcomes underscores the urgent need to address the emotional and physical well-being of women living alone. Targeted interventions and support systems are essential to alleviate the negative effects of loneliness and enhance the quality of life for this demographic.

## 5. Conclusions

This cross-sectional study revealed that, in Portugal, women in poorer health, experiencing increased fear of falling, dizziness, fatigue, anxiety, depression, and sleep issues, are more prone to feelings of loneliness, in an adverse condition such as the pandemic. These findings reinforce previous evidence indicating that the global challenges posed by the COVID-19 pandemic may have significantly impacted vulnerable populations, particularly older individuals. Future research employing longitudinal research designs, in a post-pandemic context, is warranted to explore these relationships more rigorously and to inform effective intervention development and strategies to prevent mental health problems within this population. Enhancing prevention and intervention efforts is crucial for identifying risk factors and promoting healthy aging, ultimately mitigating cognitive and physical decline in older adults.

## Figures and Tables

**Table 1:** Sample characteristics: demographic and health factors.

	*n* (%)
Gender [male]	387 (46.3)
Age, mean (sd ^1^) [min–max]	74.5 (6.7) [65–97]
How was your health before the outbreak?	
Excellent	17 (2.0)
Very good	39 (4.6)
Good	265 (31.7)
Fair	350 (41.9)
Poor	165 (19.7)
Change in your health since the outbreak	
Improved	21 (2.5)
Worsened	106 (12.7)
About the same	709 (84.8)
Diagnosed with a major health condition [yes]	163 (19.5)
Falling more often [yes]	136 (16.3)
Fear of falling [yes]	336 (40.2)
Dizziness, fainting, or blackouts [yes]	238 (28.5)
Fatigue [yes]	402 (48.1)
Takes prescription drugs regularly [yes]	768 (91.9)
Felt nervous [yes]	435 (52.0)
Sad or depressed [yes]	378 (45.2)
Trouble sleeping recently [yes]	346 (41.4)
How often do you feel lonely [often or some of the time]	279 (33.4)

^1^ sd: standard deviation; min: minimum; max: maximum.

**Table 2:** Comparison of groups according to the feelings of loneliness status.

	Feeling Lonely		
	Often or Some of the Time	Hardly Never or Never	*p*
	*n* (%)	*n* (%)	
Gender [male]	95 (34.1)	292 (52.4)	<0.001
Age, mean (sd ^1^)	74.9 (6.8)	74.4 (6.7)	0.238 ^2^
How was your health before the outbreak?			<0.001
Excellent	4 (1.4)	13 (2.3)	
Very good	8 (2.9)	31 (5.6)	
Good	64 (22.9)	201 (36.1)	
Fair	129 (46.2)	221 (39.7)	
Poor	74 (26.5)	91 (16.3)	
Change in your health since the outbreak			<0.001
Improved	6 (2.2)	16 (2.8)	
Worsened	61 (21.9)	46 (8.1)	
About the same	212 (76.0)	505 (89.1)	
Diagnosed with a major health condition [yes]	65 (23.3)	98 (17.6)	0.050
Falling more often [yes]	53 (19.0)	83 (14.9)	0.130
Fear of falling [yes]	135 (48.4)	201 (36.1)	<0.001
Dizziness, fainting, or blackouts [yes]	93 (33.3)	145 (26.0)	0.027
Fatigue [yes]	168 (60.2)	234 (42.0)	<0.001
Takes prescription drugs regularly [yes]	260 (93.2)	508 (91.2)	0.322
Feeling nervous [yes]	202 (72.4)	233 (41.8)	<0.001
Sad or depressed [yes]	197 (70.6)	181 (32.5)	<0.001
Trouble sleeping recently [yes]	153 (54.8)	193 (34.6)	<0.001

^1^ sd: standard deviation; ^2^ Independent sample *t*-test.

**Table 3:** Factors associated with feeling nervous.

	Unadjusted			Adjusted		
	OR ^1^	95% CI ^2^	*p*	OR ^1^	95% CI ^2^	*p*
Gender [female]	2.803	2.116–3.712	<0.001	2.084	1.538–2.824	<0.001
Age (z-score)	0.941	0.821–1.077	0.377	0.835	0.715–0.974	0.022
Diagnosed with a major health condition [yes]	1.503	1.061–2.129	0.022	1.297	0.883–1.906	0.185
Fatigue [yes]	2.766	2.089–3.662	<0.001	2.205	1.612–3.017	<0.001
Trouble sleeping recently [yes]	2.877	2.159–3.835	<0.001	1.989	1.456–2.717	<0.001
How often do you feel lonely [often or some of the time]?	3.648	2.671–4.983	<0.001	2.803	2.011–3.908	<0.001
R^2^ (Cox And Snell)				0.179		
R^2^ (Nagelkerke)				0.239		

^1^ OR: odds ratio; ^2^ CI: confidence interval.

**Table 4:** Factors associated with feeling sad or depressed.

	Unadjusted			Adjusted		
	OR ^1^	95% CI ^2^	*p*	OR ^1^	95% CI ^2^	*p*
Gender [female]	2.943	2.213–3.912	<0.001	2.102	1.530–2.887	<0.001
Age (z-score)	1.107	0.966–1.269	0.144	1.031	0.879–1.209	0.709
Diagnosed with a major health condition [yes]	1.925	1.361–2.724	0.022	1.747	1.177–2.593	0.006
Fatigue [yes]	2.805	2.117–3.717	<0.001	1.918	1.389–2.648	<0.001
Trouble sleeping recently [yes]	3.590	2.689–4.793	<0.001	2.636	1.914–3.630	<0.001
How often do you feel lonely [often or some of the time]?	4.991	3.651–6.823	<0.001	3.878	2.772–5.424	<0.001
R^2^ (Cox And Snell)				0.230		
R^2^ (Nagelkerke)				0.308		

^1^ OR: odds ratio; ^2^ CI: confidence interval.

## Data Availability

Data are derived from public domain resources.
